# An ultra-high density bin-map for rapid QTL mapping for tassel and ear architecture in a large F_2_ maize population

**DOI:** 10.1186/1471-2164-15-433

**Published:** 2014-06-04

**Authors:** Zongliang Chen, Baobao Wang, Xiaomei Dong, Han Liu, Longhui Ren, Jian Chen, Andrew Hauck, Weibin Song, Jinsheng Lai

**Affiliations:** State Key Laboratory of Agro-biotechnology and National Maize Improvement Center of China, China Agricultural University, Beijing, 100193 China

**Keywords:** Quantitative trait loci, Genotyping by sequencing, Next generation sequencer, Breeding, Maize

## Abstract

**Background:**

Understanding genetic control of tassel and ear architecture in maize (*Zea mays* L. ssp. *mays*) is important due to their relationship with grain yield. High resolution QTL mapping is critical for understanding the underlying molecular basis of phenotypic variation. Advanced populations, such as recombinant inbred lines, have been broadly adopted for QTL mapping; however, construction of large advanced generation crop populations is time-consuming and costly. The rapidly declining cost of genotyping due to recent advances in next-generation sequencing technologies has generated new possibilities for QTL mapping using large early generation populations.

**Results:**

A set of 708 F_2_ progeny derived from inbreds Chang7-2 and 787 were generated and genotyped by whole genome low-coverage genotyping-by-sequencing method (average 0.04×). A genetic map containing 6,533 bin-markers was constructed based on the parental SNPs and a sliding-window method, spanning a total genetic distance of 1,396 cM. The high quality and accuracy of this map was validated by the identification of two well-studied genes, *r1*, a qualitative trait locus for color of silk (chromosome 10) and *ba1* for tassel branch number (chromosome 3). Three traits of tassel and ear architecture were evaluated in this population, a total of 10 QTL were detected using a permutation-based-significance threshold, seven of which overlapped with reported QTL. Three genes (GRMZM2G316366, GRMZM2G492156 and GRMZM5G805008) encoding MADS-box domain proteins and a BTB/POZ domain protein were located in the small intervals of *qTBN5* and *qTBN7* (~800 Kb and 1.6 Mb in length, respectively) and may be involved in patterning of tassel architecture. The small physical intervals of most QTL indicate high-resolution mapping is obtainable with this method.

**Conclusions:**

We constructed an ultra-high-dentisy linkage map for the large early generation population in maize. Our study provides an efficient approach for fast detection of quantitative loci responsible for complex trait variation with high accuracy, thus helping to dissect the underlying molecular basis of phenotypic variation and accelerate improvement of crop breeding in a cost-effective fashion.

**Electronic supplementary material:**

The online version of this article (doi:10.1186/1471-2164-15-433) contains supplementary material, which is available to authorized users.

## Background

Understanding genetic control of tassel and ear architecture in maize (*Zea mays* L. ssp. *mays*) is important due to their relationship with grain yield. For efficient production of hybrid seed, inbreds used as males typically have a larger tassel that sheds greater amounts of pollen over a relatively long period of time, while the ears of the female inbred tend to be longer in length and have more kernel rows [1]. These important traits are under selection during breeding program and controlled by quantitative trait loci (QTL) [2]. QTL mapping has successfully been employed to identify relevant loci of many crucial yield-related traits in crop plants, including seed number, seed size, and plant architecture [3–6] and has been shown to be a powerful strategy to identify underlying genes and elements when combined with map-based cloning [7–9]. However, the high complexity of crop genomes and the low-coverage of genetic markers across chromosomes have posed great challenges for dissection of quantitative genetic variation by QTL analysis, especially small-effect QTL [10].

The efficiency of QTL mapping largely depends on marker density and population size. Advanced populations such as recombinant inbred lines (RILs) and nearly isogenic lines (NILs) are frequently used for QTL mapping to reduce the cost of genotyping due to the high frequency of recombination within a limited population size [10–12]. QTL mapping resolution can be improved with larger population sizes and greater marker density to detect the locations of recombination events more precisely [8]. Construction of large advanced crop populations can be both time consuming and expensive. Therefore, large populations of F_2_s, backcrosses (BC) such as BC_1_s, or other early generation crosses, combined with high through-put genotyping method provide an alternative. Vales *et al.*[13] estimated the effect of population size on QTL mapping and concluded that a large early generation population was able to detect more QTL, including small-effect QTL, than studies that used smaller advanced generation populations. A huge maize-teosinte BC_1_ population of 1749 individuals was constructed for fine mapping of QTL associated with domestication, and resulted in identification of hundreds of QTL for 22 traits [14]. When the marker density was increased in the region of the *tb1* gene, the authors were able to detect additional crossovers in the open reading frame (ORF) and flanking regions, indicating the importance of both population size and marker density for QTL cloning.

Recent advances in next-generation sequencing technologies have provided cost effective platforms for direct detection of high-quality single nucleotide polymorphisms (SNP) markers for genotyping of mapping populations [15–17]. The maize genome sequence and resources such as HapMap greatly assist mapping strategies based on high-throughput genotyping by the identifying genes and polymorphisms that may reside between intervening markers [18–21]. Genotyping-by-sequencing (GBS) [22] is a popular new method for affordably acquiring dense genome wide marker data for large sample size populations and has been successfully utilized for genetic studies in a variety of species [23–26]. Limitations of GBS include a relatively large proportion of missing data and a small, but rarely corrected, percentage of SNP genotyping sequencing errors. Recently, Spindel *et al.*[27] developed a custom-designed pipeline for SNP imputation, error correction, and streamlined data analysis based on low-coverage sequencing of a RIL population. Using imputed high-density markers, they were able to detect recombination hot and cold spots of segregation distortion with high degree of accuracy, and identify previously unreported QTL for leaf width and aluminum tolerance in rice. Another option for imputing missing SNP data is the sliding-window approach, where adjacent SNPs with same genotype in an interval are combined into bins that demarcate recombination locations across the whole population [10, 28]. The bin-map method is demonstrated to be more powerful for detecting QTL than traditional methods and has also been employed for fine mapping of yield-associated loci in rice and sorghum and root-knot nematode resistance QTL in soybean [29–31].

In this study, a large F_2_ population was generated from crossing the elite Chinese inbred Chang7-2 with the Ex-PVP line 787. The maternal line 787 is characterized by the absence of lateral tassel branches, while the paternal line used, Chang7-2, typically has about 25 branches. Dense marker data was obtained for 708 F_2_ individuals using GBS and a modified sliding-window approach, resulting in a total of 6,533 recombination bin markers. Tassel branch number, kernel row number, and ear length was measured on individual plants and used to map QTL. Our results suggest this cost effective approach is capable of rapid fine mapping QTL and candidate genes in maize.

## Results

### Sequencing, genotyping, and bin-map construction

For each F_2_ individual, the reads of the 100-bps sequences were sorted based on the indices (see Additional file 1: Table S2). A total of 551,114,523 reads with average of 755,987 reads per F_2_ individual were generated, which is equivalent to ~0.04-fold coverage of the maize genome for each F_2_ individual. The 100-mer short reads of parents and F_2_ individuals were aligned with the B73 RefGen_v2 sequence to get the physical positions of each SNP. A total of 1,155,158 high-quality SNPs were identified between two parents (~1 SNP/1.77 kb). Of these, 248,168 SNPs observed with the low coverage sequencing of the F_2_ population were selected (see Additional file 2: Figure S2, Figure S3) such that each F_2_ individual had ~15,863 SNPs, ranging from 3,371 to 33,239, and yielding a genome-wide SNP density at ∼ 1 SNP/130.3-kb per individual.

**Figure 1:**
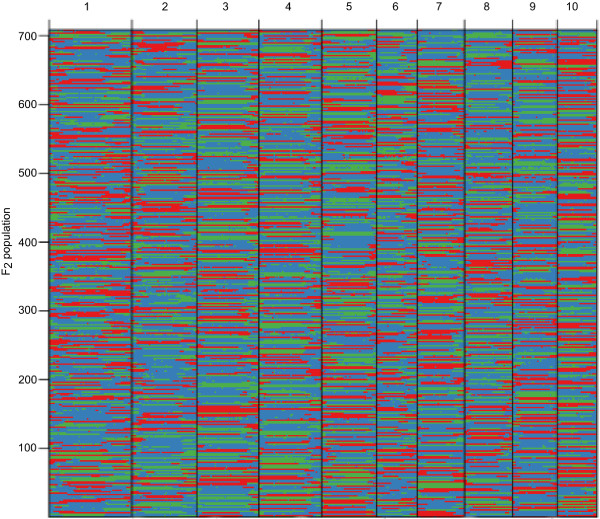
**Recombination bin-map of F**_**2**_ **population.** Bin-map consists of 6,674 bin markers inferring from 248,168 high quality SNPs in F_2_ population. Physical position is based on B73 RefGen V2 sequence. Red: Chang7-2 genotype; Green: 787 genotype; Blue: heterozygote.

The recombination maps were divided into skeleton bins [32] for further genetic analysis, then grouped into the 6,674 bin markers as described in method (Figure 1). The length of bin markers ranged from 100 Kb to 3.3 Mb, with a mean of 279.5 Kb, and a median of 200 Kb. In total, 88.3% of bin markers were less than 0.5 Mb in length. There were 219 bins larger than 1.0 Mb in size and 3 big bins of more than 3.0 Mb dispersed on chromosomes 2 (Bin2_322) and 8 (Bin8_158 and Bin8_1) (see Additional file 2: Figure S4). The number of crossovers for each individual was calculated based on the bin markers. The average number of crossovers was 26.3, and ranged from 10 to 65 with median of 24 (see Additional file 2: Figure S5).

For construction of the genetic linkage map, segregation ratios of each bin marker were calculated and 141 out of 6674 bin markers were highly distorted from 1:2:1 (Chi-squared test, *P* < 10^-10^) (see Additional file 2: Figure S6). Distorted markers were considered to be related to difficulties in genotyping and deleted. The total distance of the genetic map was 1,396.0 cM, approximately 0.2 cM per bin, and the average distance between neighboring bin markers ranged from 0.1 cM to 11.5 cM (see Additional file 2: Figure S7).

### The quality and accuracy of the map

**Figure 2:**
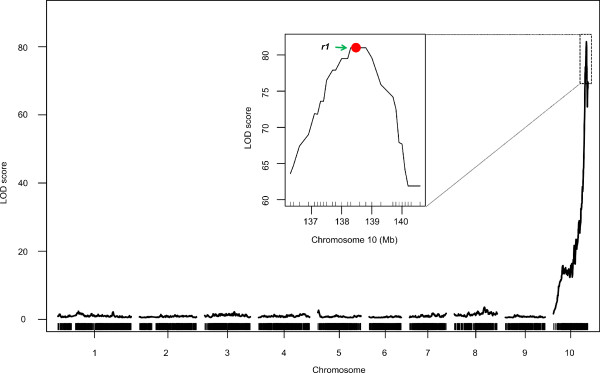
**Mapping of QTL controlling color of silk in F**_**2**_ **population and the location of** ***r1*** . Curves in plot indicate the genetic or physical coordinate (X-axis) and LOD score (Y-axis) of detected QTL. Mapping curve of QTL controlling color of silk locates on chromosome 10; the box inside is the zoom-in image of the peak on chromosome 10. Red dot presents the relative physical position of *r1* gene.

In order to examine the mapping power of the strategy with highly heritable traits, we recorded the color of silks (COS) for the 611 individuals with an ear. The color was divided into two groups with red and green and the phenotype was mapped with the *scanone* function in R/qtl. Only one QTL was detected, but the peak encompassed the cloned gene *colored 1* (*r1*) [33] at Bin10_460 with a high LOD score of 81 (Figure 2).

Dominant *R1* controls the pigmentation of kernels and other plant tissues, including silks, by regulating the genes of enzymes involved in anthocyanin biosynthesis. Chang7-2 and 484 F_2_s had red silks while the remaining individuals and 787 had green silks. The ratio of red silk versus green silk was close to 3:1 (Chi-squared test, *P* = 0.016), suggesting COS was mostly controlled by a single dominate gene. The confidence interval of the QTL spanned ~700-kb and contained three bins, but these results demonstrate the high mapping resolution of the bin-map in combination with large population size.

### QTL mapping for tassel and ear architecture

**Figure 3:**
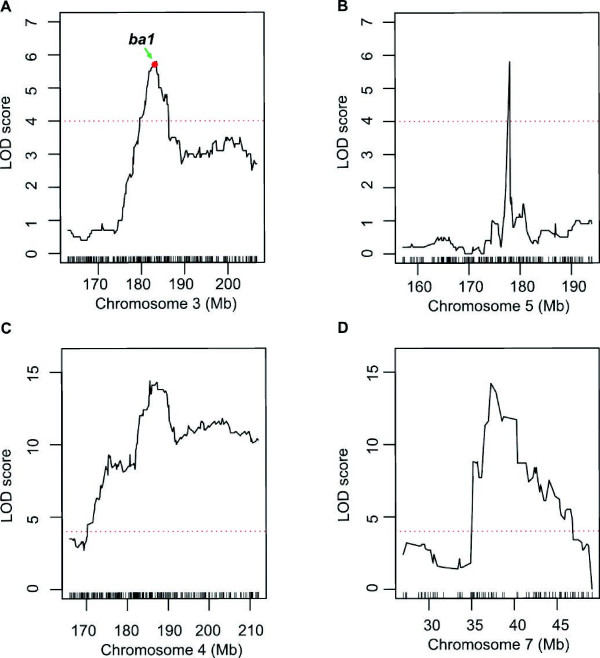
**Mapping of QTL controlling tassel branch number in F**_**2**_ **population and the location of** ***ba1*** **. (A-** **D)** Curves in plot indicate the physical coordinate (X-axis) of bin markers and LOD score (Y-axis) of detected QTL in chromosomes 3, 4, 5 and 7, and precise location of QTL for tassel branch number on chromosome 3 harboring a cloned gene (*ba1*); red dot presents the relative physical position of *ba1* gene. Red dot lines present the LOD threshold.

Based on a permutation determined LOD threshold of about 4, ten QTL in total were identified: seven for tassel branch number on chromosomes 1, 3, 4, 5, 7, 8 and 9, one for kernel row number on chromosome 5, and two QTL for ear length on chromosomes 4 and 5 (Table 1). The QTL with the largest effect on tassel branch number were *qTBN4* and *qTBN7*, where alleles from 787 decreased the number of tassel branches by 2.1 and 5.9, explaining 6.2% and 6.3% of phenotypic variation, respectively. *qTBN4* spanned a genetic distance of about 7 cM, corresponding to a physical distance of about 6.1 Mb in B73 RefGen_v2 genome, and *qTBN7* spanned a genetic distance of about 0.6 cM, corresponding to a physical distance of about 1.6 Mb (Table 1, Figure 3C and D). The location of *qTBN4* and *qTBN7* overlapped with previously reported QTL, i.e. *qTBN4* versus *TBN4.100w* and *qTBN7* versus *TBN7.34f* and *TBN7.43w*[1, 14]. However, mapping with this population narrowed down the intervals to 6.1 Mb and 1.6 Mb on chromosomes 4 and 7, respectively. *qTBN3* explained 2.6% of the phenotypic variation and mapped to a region between Bin3_628 and Bin3_657, a physical distance of 5.5 Mb (Figure 3A). *barren stalk1* (*ba1*), which is involved in the patterning of branches and inflorescence structures in maize [34], is located in the bin of the QTL peak (Bin3_641). The physical interval of *qTBN5* was the smallest one among the identified QTL at ~800 Kb in length (Figure 3B; Table 1); and the phenotypic variation explained by this locus was 2.3%. *qTBN1* was located in a large interval of 56.6 Mb on chromosome 1, and explained 2.1% of the phenotypic variation. Both of *qTBN1* and *qTBN5* were consistent with *TBN1.112w* and *TBN5.105w* in a report by Briggs *et al.*[14], respectively. The remaining two previously unreported QTL (*qTBN8* and *qTBN9*) had an effect size of ~1 branch, with Chang7-2 carrying the alleles for greater branch number (Table 1).

**Table 1:** **QTL identified for three traits using high-density SNP bin-map**

Trait	QTL	Chr	Bin	Position (cM)	Position (Mb)	Interval (Mb)^***a***^	Physical length (Mb)	LOD score	Additive^***b***^	Dominance	R^2^ (%)^***c***^	Published QTL/gene
TBN	*qTBN1*	1	Bin1_734	175.41	199.1	194.3-250.9	56.6	4.9	1.1	0.7	2.1	*TBN1.112w*
	*qTBN3*	3	Bin3_641	120.23	183.5	180.8-186.3	5.5	6.0	-1.2	0.4	2.6	*ba1*
	*qTBN4*	4	Bin4_663	145.68	185.6	183.9-190.0	6.1	13.8	-2.1	-0.2	6.2	*TBN4.100w*
	*qTBN5*	5	Bin5_602	118.37	178.0	177.2-178.0	0.8	5.8	-1.2	0.7	2.3	*TBN5.105w*
	*qTBN7*	7	Bin7_146	39.00	37.3	37.0-38.6	1.6	14.2	-5.9	-2.1	6.3	*TBN7.34f, TBN7.43w*
	*qTBN8*	8	Bin8_1	3.30	3.3	0.0-7.6	7.6	4.6	-1.1	-0.4	2.0	None
	*qTBN9*	9	Bin9_283	73.00	74.5	44.4-74.9	30.5	7.3	-1	-0.1	3.0	None
KRN	*qKRN5*	5	Bin5_588	114.29	174.6	173.5-178.3	4.8	5.7	-0.7	-0.1	5.7	*qkrow2*
EL	*qEL4*	4	Bin4_650	140.63	182.9	182.2-188.1	5.9	9.4	-1.3	0.4	6.8	*qearl24*
	*qEL5*	5	Bin5_659	134.99	194.0	186.9-194.0	7.1	5.9	-0.9	0.4	4.1	None

TBN tassel branch number; KRN Kernel row number; EL ear length.

^*a*^1.5-LOD support interval of the QTL.

^*b*^Additive effect: positive values of the additive effect indicate that alleles from Chang7-2 were in the direction of increasing trait score.

^*c*^Percentage of the phenotypic variation explained by the QTL.

Only one small-effect QTL for kernel row number was identified in our large F_2_ population (Table 1), which explained 5.7% of the phenotypic variation. This QTL on chromosome 5, *qKRN5*, is in the similar location as *qkrow2*, which was detected in a small F_4_ population [35], but our locus was mapped to a smaller physical interval of 4.8 Mb. For ear length, two QTL on chromosomes 4 and 5 were detected, explaining a total of 10.9% of the phenotypic variation (Table 1). Austin and Lee [36] identified an ear length QTL with a large confidence interval in genetic bin 4.08, but the QTL identified in this study was narrowed down to a 5.9 Mb region defined by Bin5_637 and Bin5_659.

### Candidate gene prediction

The small physical intervals of *qTBN7* and *qTBN5* encompassed only 13 and 17 protein coding genes, respectively (Table 1; Additional file 2: Table S1), according to maize gene annotation database accessible at MaizeGDB (http://www.maizegdb.org). Recent work in Arabidopsis and maize have showed that MADS-box TFs are possible co-factors of LEAFY transcriptional factor in promoting floral differentiation [37, 38]. In addition, BTB/POZ domain proteins (BPMs) physically recognize and target a specific subclade of class I homeobox-leucine zipper (HD-ZIP) transcription factors for proteosomal degradation [39]. These were found to be positively modulated by RA1 and/or KN1, which are involved in floral organ development in maize [38]. Among the candidate genes in the intervals of *qTBN5* and *qTBN7*, two genes (GRMZM2G316366, GRMZM2G492156) are MADS-box transcription factors and one gene (GRMZM5G805008) encodes a BTB/POZ domain protein (BPMs).

## Discussion

### Advantages of the GBS based bin-map strategy

Genotyping by sequencing was developed to reduce the complexity of diverse large genomes for SNP discovery and genotyping of given populations. It captures the interest of geneticists and breeders because of the advantages: (1) no prior knowledge of genome information is required, as sequence polymorphisms are detected in the process of genotyping a population, though parental SNP information is still needed for assigning genotypes (2) the use of 4 to 8-base barcode adapters allows parallel sequencing of hundreds of DNA samples, dramatically reducing the time and labor required for genotyping; (3) methylation-sensitive restriction enzymes effectively filter repetitive sequences thus to simplify the complexity of genomes for downstream bioinformatics analysis; (4) the genome-wide approach of SNP detection provides a dense distribution of markers across chromosomes, which is superior to traditional PCR-based markers.

Unequal recovery of restriction-enzyme-cut fragments among samples combined with a high level of sample multiplexing can result in sparse marker data when conducting GBS. This problem can be solved by simply reducing the amount of sample multiplexing. However, cost is prime consideration when genotyping large populations. SNP imputation using sparse data is a typical compromise that has been effectively employed. It is quite impossible to perform SNP imputation based on sparse SNP markers with a lot of missing data for species lacking a reference genome information, but GBS can still be utilized to generate relatively low density markers by a technique known as restriction site associated DNA sequencing (RAD) and the tagged SNPs used for QTL analysis and genomic selection [40, 41]. In species with a reference genome, SNP imputation can be done with high accuracy in biparental mapping populations [22]. The bin-map strategy was demonstrated to be efficient in generation of ultra high-density of bin markers based on sparse SNP information and identification of QTL with high resolution in rice and sorghum [10, 29]. Compared with conventional molecular marker, RFLP/SSR or single SNP markers, bin markers are the most informative and parsimonious set for a given population. Here, we took advantage of the GBS protocol to lower cost and labor of genotyping of a large F_2_ population, generated abundant parental SNP information, and utilized the maize reference genome for SNP calling and imputation. In our F_2_ population, the number of crossovers for each individual was around 26 (Additional file 2: Figure S1), indicating that about 2 to 3 recombination events occurred per chromosome. The limited recombination in F_2_s allows for precise imputation of missing SNPs within long range regions using the bin-map strategy. In total, we obtained 6,533 high confidence bin markers. The length of bin markers ranged from 0.1 Mb to 3.3 Mb with a mean of 279.5-Kb, suggesting that a QTL could be narrowed down to a small interval harboring dozens of genes or less.

### Superiority of QTL mapping in large early generation populations

Genetic variations, including artificial mutagenesis and naturally occurring variation, help dissect the molecular basis of many agronomically important traits in crops. Mutagenesis populations such as the EMS mutagenesis population [42], *Ac-Ds* induced population [43] and *Mutator*-mutagensis population [44], have helped to isolate serials of genes controlling inflorescence development [34, 45, 46]. Crop breeding is largely dependent on the quantitative genetic variation among germplasm. QTL mapping and genome-wide association analysis (GWAS) are two dominant strategies to analyze the natural variants. However, development of a GWAS population requires highly diverse germplasm and an ultra-high-density SNP map to capture as many of the historical recombinant events as possible [47], which is not so easy for every laboratory. Developing, genotyping, and phenotyping advanced generation QTL mapping populations, such as RILs or NILs, with traditional methods is a very costly and time consuming process for crop species. QTL mapping performed with F_2_, F_2:3_ or BC_1_ populations has been limited by population size and sparse genetic maps. Mapping resolution in early generation populations using our method may be further improved by increasing population size or recombining the material for an additional mating generation. High-resolution genetic maps capture the location of every recombination event and make full use of the linkage information in the population. Therefore, we generated a large F_2_ population and constructed an ultra-high-density genetic bin-map to capture as many recombinant events as possible. Among the QTL we detected, the physical intervals of *qTBN5* and *qTBN7* were ~800-Kb and 1.6 Mb, respectively (Table 1), suggesting high efficiency in identification of QTL with this approach. A previous study using a large maize-teosinte BC_1_ population with 1749 individuals identified several recombination events within gene body of the teosinte branch QTL-candidate gene *tb1* [14]. We envisage that QTL mapping with large early generation populations (~2000 individuals) and high-density genetic maps may be able to narrow confidence intervals for major QTL down to single genes.

Quantitative traits have complex genetic regulation and often interact with the environment. With our method, we were able to identify QTL in regions with previously reported loci for relevant traits mapped in different populations, but with a superior interval size and a single environment. In maize, tassel branch number is a component of tassel architecture involving a subset of inflorescence patterning genes, whose effects are known to be strongly influenced by the environments. Briggs, *et al.*[14] showed that a few of QTL for tassel branch number detected in two different environments were overlapped (5 pairs out of 33 QTL) using an extremely large BC_1_ population. For flowering time, *ZmCCT* (or *POLL10.47w*), a large-effect QTL, was detectable by that study only in one environment [14], and identified again in a subsequent study using the derived BC2S3 RIL population [8]. Takagi *et al.*[48] proposed a fast QTL mapping method called QTL-seq using whole genome resequencing of two bulked populations that have opposite and extreme phenotypes. With this approach, the authors identified QTL and candidate genes for rice seedling vigor using an F_2_ population without replication that were able to be validated using an F_7_ RILs population. Although it is preferable to identify QTL with several replications in different environments, detection of QTL without replication is a reasonable alternative approach if narrow interval loci can be identified cost-effectively. Furthermore, most QTL in this study overlapped with regions previously identified in other studies using different mapping populations, which provides additional confidence in the validity of the results. Reduction of the QTL interval with large populations and dense marker maps for fine mapping is valuable in this context for better defining candidate genes underlying mapped loci. F_2:3_ families are often used to assess the phenotype of F_2_ individuals with replication, but QTL detected by F_2_ and F_2:3_ populations are expected to overlap. Therefore, in view of quick mapping of useful QTL for complex traits, high-density markers combined with larger population size, and QTL-seq method in early-generation populations are good options for improving the traditional mapping approach. Another strategy using rapid mapping to examine QTL by environment effects would be to divide the large population between environments and augment with replicated checks to adjust for the environmental effects.

### Accuracy of the bin-map in mapping QTL

The quality and accuracy of the bin-map for QTL detection was verified by the mapping of two known genes: *R1*, a qualitative locus that controls the color of silk, and *ba1*, which regulates initiation of lateral branches of the tassel.

Formation of anthocyanin in maize plant tissues and kernels was intensively studied in last century. Five functional genes (*C2, A1, A2, Bz1* and *Bz2*) encoding enzymes of anthocyanin synthesis and four regulatory genes (*R1, B, C1* and *Pl1*) are known to control pigmentation of plant tissues and seeds [49]. R1 and B are bHLH transcription factors, which involve in regulation of anthocyanin synthetic genes by cooperation with C1 or PL1, the R2R3-MYB transcription factors [50]. In this study, we detected a high LOD score of 81 in chromosome 10 corresponding to *R1* allele at Bin10_460, which was located at the peak of the QTL spanning 700-Kb in length, demonstrating the high accuracy of the bin-map.

Tassel branching is determined by lateral meristems and many genes including *lg2, ba1, spi1, ra1, ra2* and *ra3* have been reported to be involved in determination of the tassel branch number [2, 38, 51, 52]. *ba1* (*barren stalk1*) is a bHLH transcriptional factor that participates in initiation of all aerial lateral meristems [34]. Analysis of nucleotide diversity in distinct regions of *ba1* among inbred lines, landraces and teosintes has showed that *ba1* was under selection during the breeding program of modern maize, indicating its historical usefulness in maize improvement [34]. Our mapping results for tassel branch number identified Bin3_641 as the peak of *qTBN3* on chromosome 3, which harbors the *ba1* gene and indicates the ability of the method to rapidly fine map gene regions with important contributions to the expression of complex quantitative traits.

## Conclusions

We demonstrated that use of a high-density genetic map combined with large population size and an early generation population is able to improve mapping efficiency in QTL analysis. In view of the abundance of useful naturally occurring variation in germplasm and extremely low cost of sequencing-based genotyping, we propose that QTL mapping in large early generation populations derived from bi-parental crosses is a highly efficient method for rapid identification of useful alleles. This will help to dissect the molecular mechanisms underlying important traits and accelerate crop improvement in a cost-effective fashion by reducing the time required for effective genetic mapping in crops.

## Methods

### Plant materials and phenotyping

An F_2_ population consisting of 708 individuals was derived from the selfed cross of maize inbred lines Chang7-2 as male parent and 787 as female parent. Chang7-2 is a parental line for the Chinese elite hybrid Zhengdan958, which is widely cultivated in China. 787 is an Ex-PVP line produced by five generations of full-sib recurrent selection and subsequent selfing from a cross of U.S. public varieties VA17 and VA29, where VA17 is descended from WF9/T8 and VA29 from the open pollenated variety ‘Golden Queen’. The tassel branch number (TBN) of Chang7-2 is 25.5 ± 1.5, whereas 787 is distinguished by an absence of branching and only has a central spike. Ear length (EL) and kernel row number (KRN) are similar between Chang7-2 and 787, but Chang7-2 has red colored silks. Phenotypic data for tassel branch number, kernel row number, ear length and silk color was collected on individual F_2_ plants grown in a field trial in 2012 at the experimental farm of China Agricultural University in Beijing, China (see Additional file 2: Figure S1). The color of silk (COS) was recorded based on the following scale: red and pale-red as 1, green as 0. In total, 692 individuals were evaluated for tassel branch number, 550 for ear length, and 462 for kernel row number, due to losses resulting from bareness, insect damage, disease, or in the case of row number, irregular ears.

### DNA extraction and genotyping by sequencing

Genomic DNA from the F_2_ population and parents was obtained by using a urea–chloroform-phenol based extraction method [53] on 100 mg fresh leaf tissue after freezing in liquid nitrogen and grinding. Genotyping by sequencing, as described by Elshire *et al.*[22], was used for high-throughput sequencing of the F_2_ individuals using *Ape*KI (New England Biolabs, Ipswitch, MA) for fragmentation and 240 digested DNA samples distinguished with 4 to 8 bases of barcode adapter indices (see Additional file 1: Table S2) were combined and purified using a QIAquick PCR Purification Kit (Qiagen, Valencia, CA). The ligation products from each library were amplified using a Phusion® High-Fidelity PCR Kit (New England Biolabs, Ipswitch, MA) in 50 μL volumes containing the following primers: (A) 5′-AATGATACGGCGACCACCGAGATCTACACTCTTTCCCTACACGACGCTCTTCCGATCT and (B) 5′-CAAGCAGAAGACGGCATACGAGATCGGTCTCGGCATTCCTGCTGAACCGCTCTTCCGATCT. DNA fragments from libraries between 170–350 bps were thus enriched and ready for next-generation sequencing by an Illumina Hiseq2000 sequencer. The genome of parental lines, Chang7-2 and 787, were directly sequenced by Illumina Hiseq2000 to ~27× coverage (accession number, NCBI: SRX120903) and ~1× coverage (accession number, NCBI: SRX122168), respectively [54]. The raw reads were sorted according to indices, and the high-quality SNPs between parents were called by alignment with B73 RefGen_v2 sequence (http://www.maizegdb.org) using BWA package [20, 55] and Genome Analysis Toolkit (GATK) [56].

### Genotyping and bin-map construction

High-density genetic maps of populations with high linkage disequilibrium contain many redundant markers that provide no new information, but increase the computational requirements of mapping. Furthermore, a small percentage of genotypes are falsely called due to sequencing error. To address these issues, a modified version of the sliding-window approach developed by Huang *et al.*[28] was applied. Genotypic data was scanned with a window size of 18 SNPs and a step size of 2. For each individual, the ratio of SNP alleles from Chang7-2 and 787 within the window was calculated. Windows with 15 or more SNPs from either parent were considered to be homozygous for an individual, while those with less were classified as heterozygous. Adjacent windows with same genotypes were combined into blocks and the recombinant breakpoints were assumed to be at the boundary of adjacent blocks with different genotypes. Next, a bin-map was generated by aligning and comparing the genotypic maps of individual F_2_s over 100-kb intervals. Consecutive 100-kb intervals that lacked a recombination event within the population were joined into bins and the bins used as markers. For construction of the linkage map, bin markers which were nearly monomorphic or had only two genotypes across all 708 individuals were removed and the map was constructed using Haldane’s equation and the *est.map* function of the R/qtl package [57]. The same package was used for multiple-QTL mapping (MQM). The LOD threshold was determined using 1000 permutations and a threshold of *P ≤* 0.05. Other analyses of phenotypic data, along with figures and graphs were constructed using R as well.

### Availability of supporting data

The data set supporting the results of this article is available in the Sequence Read Archive (http://www.ncbi.nlm.nih.gov/sra/) with the accession number 'SRP042173'. All data sets supporting the results of this article are included within the article.

## Electronic supplementary material

Additional file 1: Figure S1: Variation of phenotypic traits in F_2_ individuals. (A) tassel branch number, the number of tassel branch ranged from 1 to 31, with mean of 8.1 and median of 7; (B) earl length, the earl length ranged from 6 to 25 cm with average of 15.3 cm and median of 15.5 cm; (C) kernel row number, the number of kernel row ranged from 12 to 22, with mean of 16.2 and median of 16. **Figure S2.** Chromosome summary of high quality SNPs number. SNP were identified from high-coverage sequences of Chang 7-2 and 787 and low-coverage sequences of 708 F_2_ individuals. Blue bars indicate SNPs identified between two parents; red bars indicate SNPs in 708 F_2_ population. **Figure S3.** Distribution of 248,168 high quality SNPs identified from low-coverage sequences of 708 F_2_ individuals. The physical positions on each chromosome are based on B73 RefGen_v2 sequence. The short blue lines indicate the SNP density (SNPs/500-kb). The red point on each chromosome indicates the centromere. **Figure S4.** The distribution of bin marker length. **Figure S5.** The number of crossover in each F_2_ individual. The number of crossover ranged from 10 to 65, with average of 26.3 and median of 24. Blue dot line indicates the mean of crossover. **Figure S6.** The ratio of three genotypes for each bin marker. (A) Negative log_10_(*P*) values of the chi-test of the ratios; (B) the proportions of genotypes for each bin markers. AA: homozygous Chang7-2, AB: heterozygote and BB: homozygous 787. **Figure S7.** Comparison of physical map with genetic map of 6533 bin markers. The order of the bin markers were depended on the physical position of each marker. The left lines of ladder-shaped boxes represented the physical map, and the right lines indicated the genetic map. **Table S1.** Genes located in the intervals of *qTBN5* and *qTBN7*. (DOC 875 KB)

Additional file 2: Table S2: The 4 to 8-base barcode sequences used in parallel sequencing of F_2_ population. (XLSX 17 KB)
