# Supplementary material for: An ultra-high density bin-map for rapid QTL mapping for tassel and ear architecture in a large F2 maize population
Source: BMC Genomics. 2014 Jun 4;15(1):433. doi: 10.1186/1471-2164-15-433 (PMC4059873; doi:10.1186/1471-2164-15-433)
Supplement: Supplementary file 1 — Additional file 1: Figure S1: Variation of phenotypic traits in F2 individuals. (A) tassel branch number, the number of tassel branch ranged from 1 to 31, with mean of 8.1 and median of 7; (B) earl length, the earl length ranged from 6 to 25 cm with average of 15.3 cm and median of 15.5 cm; (C) kernel row number, the number of kernel row ranged from 12 to 22, with mean of 16.2 and median of 16. Figure S2. Chromosome summary of high quality SNPs number. SNP were identified from high-coverage sequences of Chang 7-2 and 787 and low-coverage sequences of 708 F2 individuals. Blue bars indicate SNPs identified between two parents; red bars indicate SNPs in 708 F2 population. Figure S3. Distribution of 248,168 high quality SNPs identified from low-coverage sequences of 708 F2 individuals. The physical positions on each chromosome are based on B73 RefGen_v2 sequence. The short blue lines indicate the SNP density (SNPs/500-kb). The red point on each chromosome indicates the centromere. Figure S4. The distribution of bin marker length. Figure S5. The number of crossover in each F2 individual. The number of crossover ranged from 10 to 65, with average of 26.3 and median of 24. Blue dot line indicates the mean of crossover. Figure S6. The ratio of three genotypes for each bin marker. (A) Negative log10(P) values of the chi-test of the ratios; (B) the proportions of genotypes for each bin markers. AA: homozygous Chang7-2, AB: heterozygote and BB: homozygous 787. Figure S7. Comparison of physical map with genetic map of 6533 bin markers. The order of the bin markers were depended on the physical position of each marker. The left lines of ladder-shaped boxes represented the physical map, and the right lines indicated the genetic map. Table S1. Genes located in the intervals of qTBN5 and qTBN7. (DOC 875 KB) [file 12864_2014_6116_MOESM1_ESM.doc]

**SUPPORTING INFORMATION**

**Figure S1. Variation of phenotypic traits in F_2_ individuals.** (A) tassel branch number, the number of tassel branch ranged from 1 to 31, with mean of 8.1 and median of 7; (B) earl length, the earl length ranged from 6 to 25 with average of 15.3 and median of 15.5; (C) kernel row number, the number of kernel row ranged from 12 to 22, with mean of 16.2 and median of 16.

**Figure S2. Chromosome summary of high quality SNPs number.** SNP were identified from high-coverage sequences of Chang 7-2 and 787 and low-coverage sequences of 708 F_2_ individuals. Blue bars indicate SNPs identified between two parents; red bars indicate SNPs in 708 F_2_ population.

**Figure S3. Distribution of 248,168 high quality SNPs identified from low-coverage sequences of 708 F_2_ individuals.** The physical positions on each chromosome are based on B73 RefGen_v2 sequence. The short blue lines indicate the SNP density (SNPs/500-kb). The red point on each chromosome indicates the centromere.

**Figure S4. The distribution of bin marker length.**

**Figure S5. The number of crossover in each F_2_ individual**. The number of crossover ranged from 10 to 65, with average of 26.3 and median of 24. Blue dot line indicates the mean of crossover.

**Figure S6. The ratio of three genotypes for each bin marker.** (A) Negative log_10_(*P*) values of the chi-test of the ratios; (B) the proportions of genotypes for each bin markers. AA: homozygous Chang 7-2, AB: heterozygote and BB: homozygous 787.

**Figure S7. Comparison of physical map with genetic map of 6533 bin markers**. The order of the bin markers were depended on the physical position of each marker. The left lines of ladder-shaped boxes represented the physical map, and the right lines indicated the genetic map.

**Table S1. Genes located in the intervals of *qTBN5* and *qTBN7*.**

**Table S2. The 4 to 8-base barcode sequences used in parallel sequencing of F2 population.**


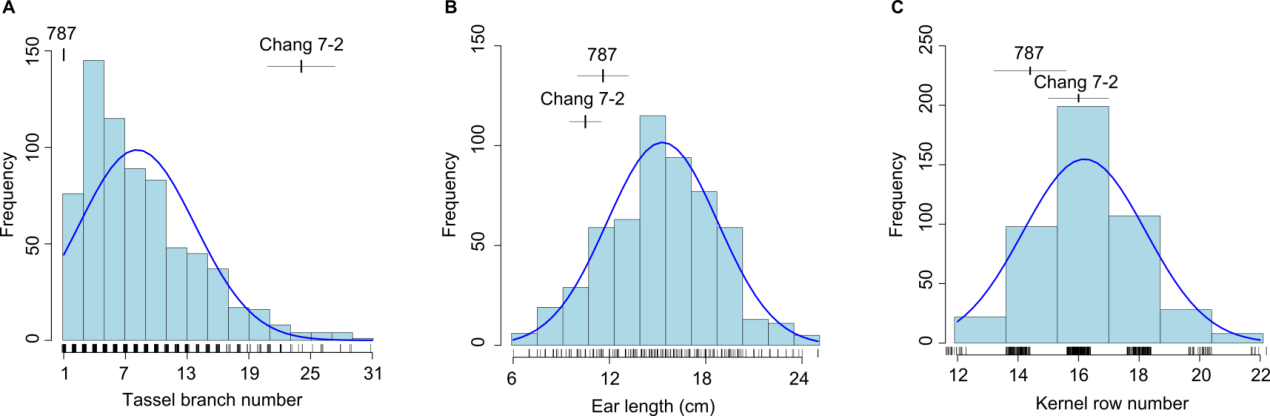


**Figure S1. Variation of phenotypic traits in F_2_ individuals.** (A) tassel branch number, the number of tassel branch ranged from 1 to 31, with mean of 8.1 and median of 7; (B) earl length, the earl length ranged from 6 to 25 with average of 15.3 and median of 15.5; (C) kernel row number, the number of kernel row ranged from 12 to 22, with mean of 16.2 and median of 16.


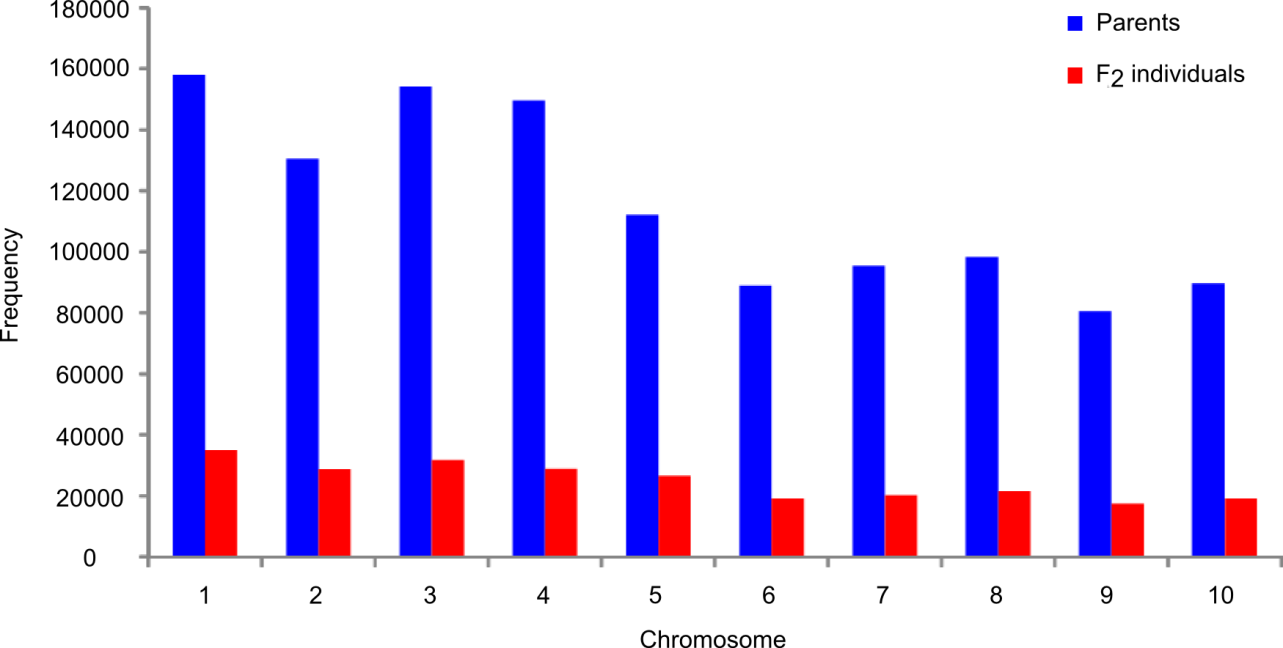


**Figure S2. Chromosome summary of high quality SNPs number.** SNP were identified from high-coverage sequences of Chang 7-2 and 787 and low-coverage sequences of 708 F_2_ individuals. Blue bars indicate SNPs identified between two parents; red bars indicate SNPs in 708 F_2_ population.


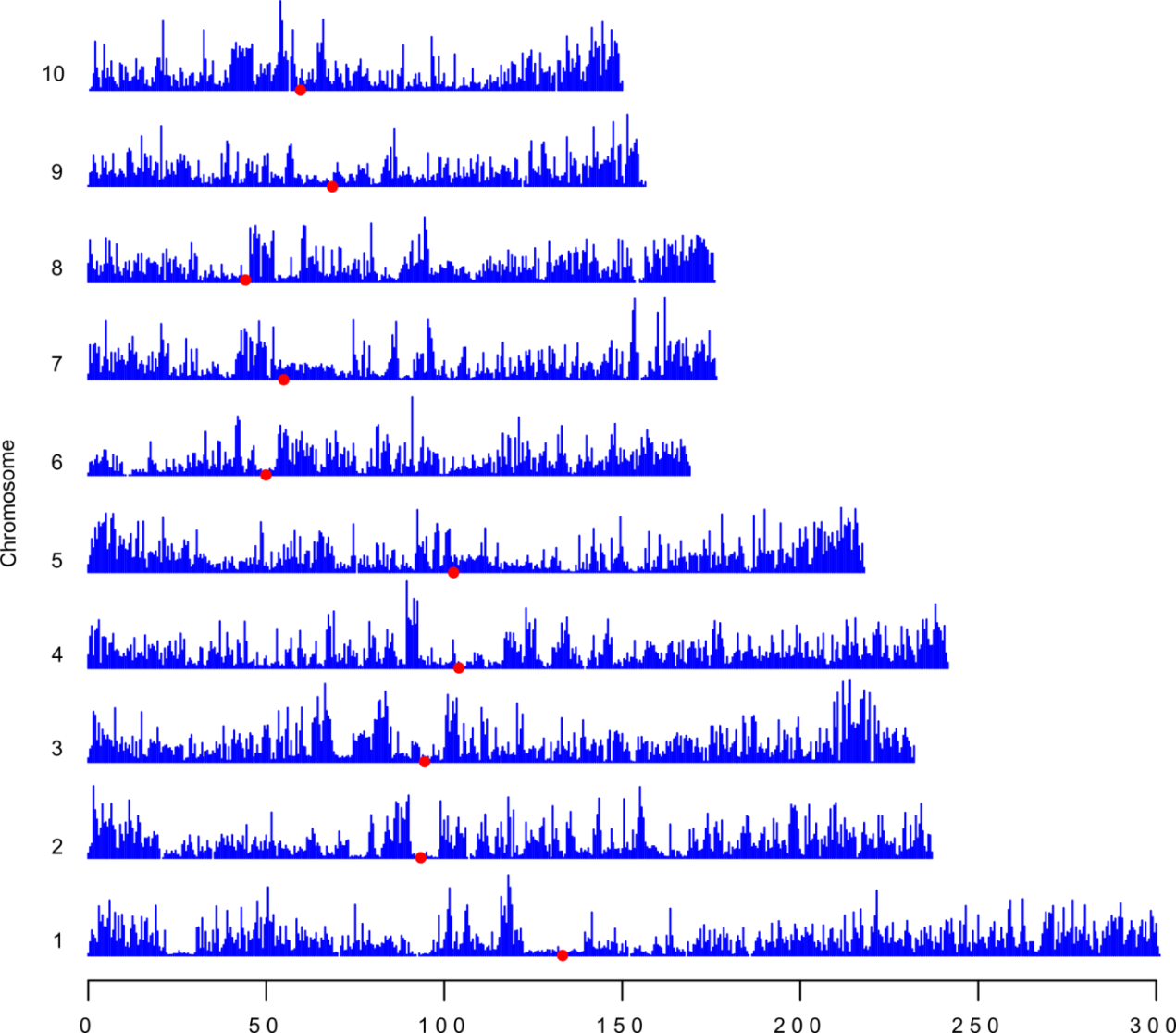


**Figure S3. Distribution of 248,168 high quality SNPs identified from low-coverage sequences of 708 F_2_ individuals.** The physical positions on each chromosome are based on B73 RefGen_v2 sequence. The short blue lines indicate the SNP density (SNPs/500-kb). The red point on each chromosome indicates the centromere.


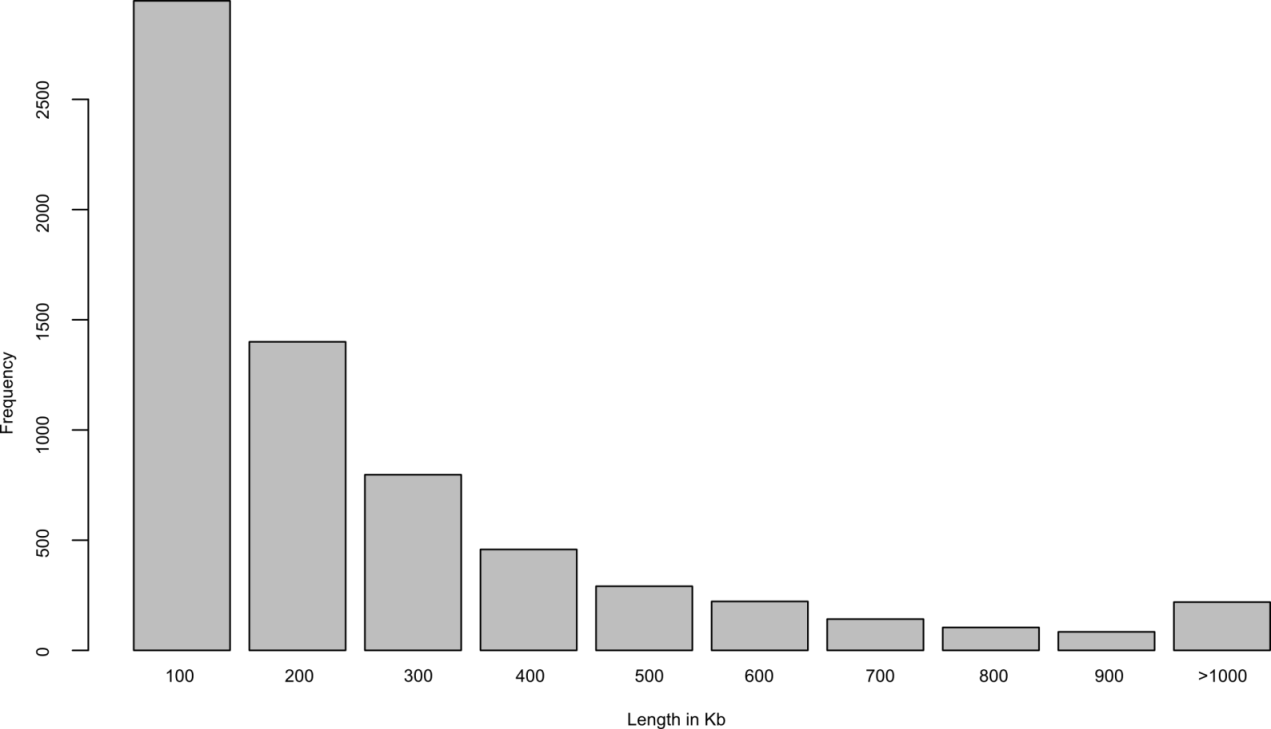


**Figure S4. The distribution of bin marker length.**


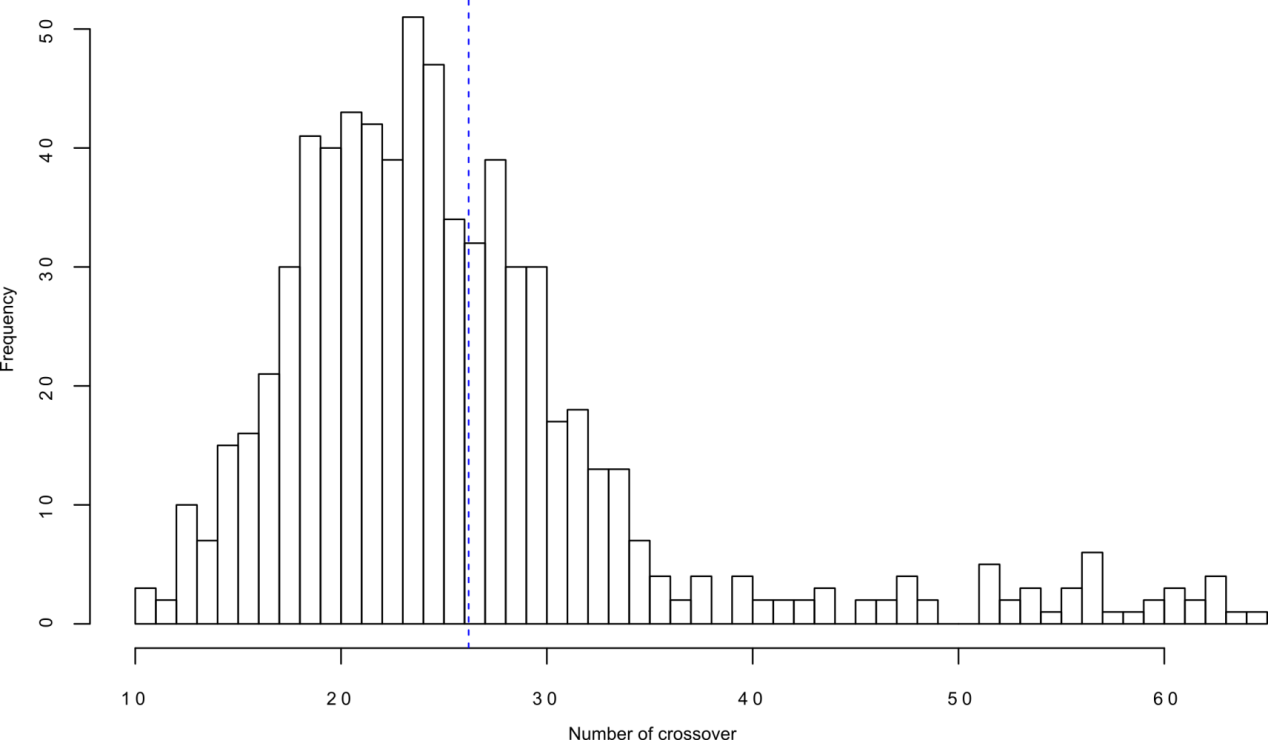


**Figure S5. The number of crossover in each F_2_ individual**. The number of crossover ranged from 10 to 65, with average of 26.3 and median of 24. Blue dot line indicates the mean of crossover.


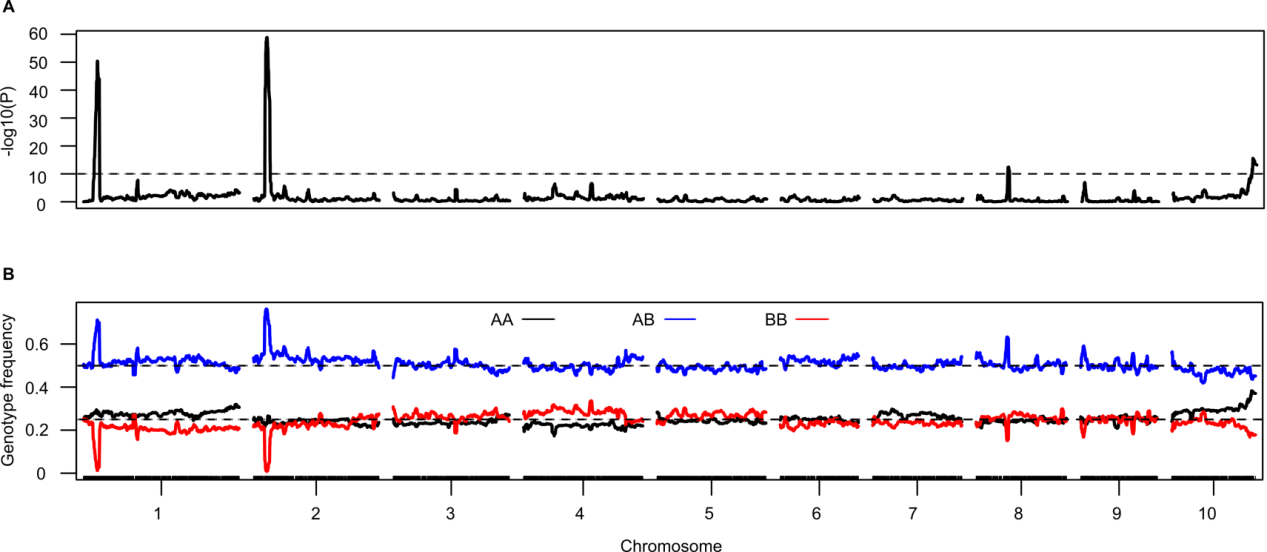


**Figure S6. The ratio of three genotypes for each bin marker.** (A) negative log_10_(*P*) values of the chi-test of the ratios; (B) the proportions of genotypes for each bin markers. AA: homozygous Chang 7-2, AB: heterozygote and BB: homozygous 787.


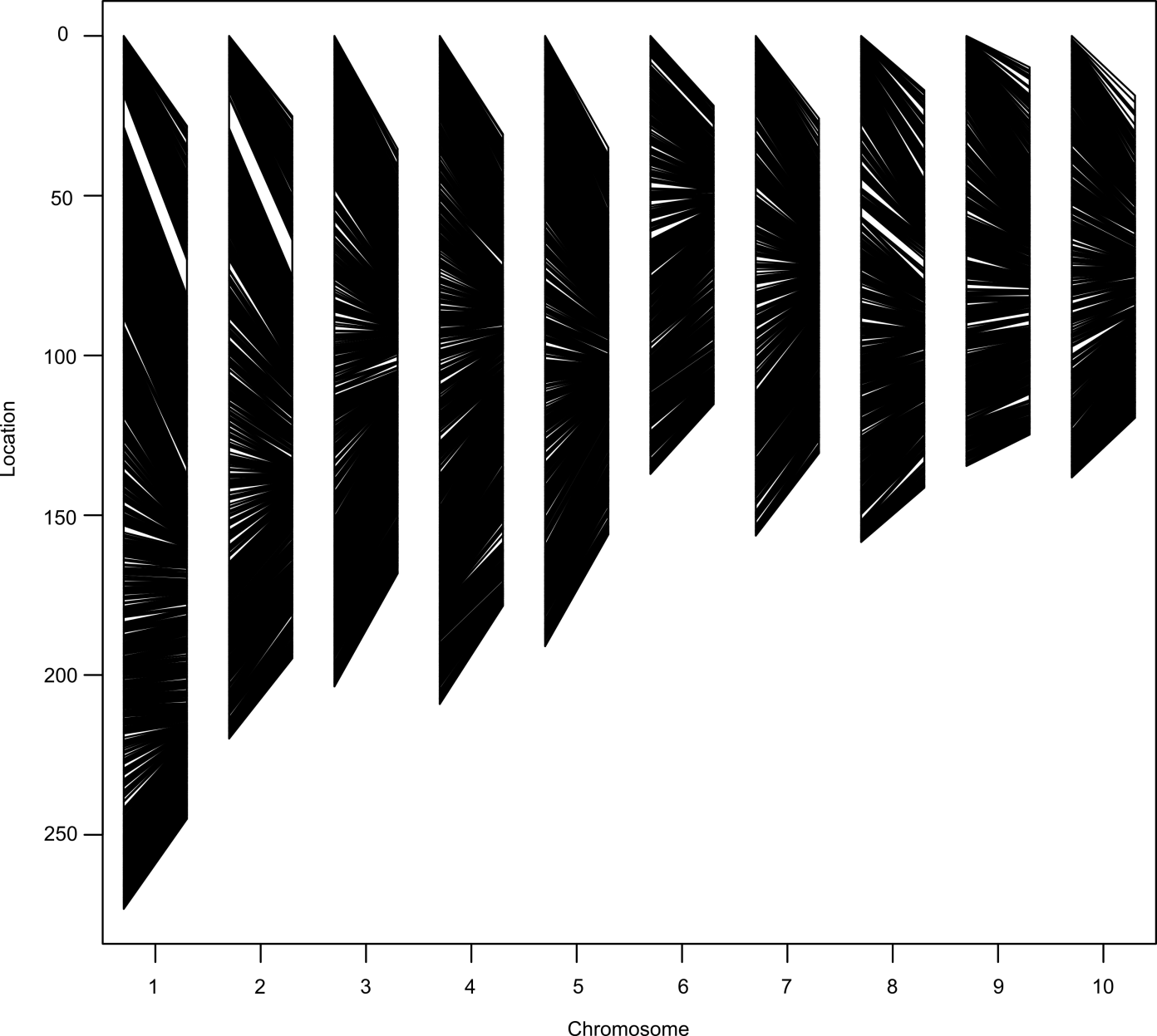


**Figure S7. Comparison of physical map with genetic map of 6533 bin markers**. The order of the bin markers were depended on the physical position of each markers. The left lines of ladder-shaped boxes represented the physical map, and the right lines indicated the genetic map.

**Table S1. Genes located in the intervals of *qTBN5* and *qTBN7*.**

| Chr. | Pos_start | Pos_end | Gene ID | Annotation |
| --- | --- | --- | --- | --- |
| 5 | 177222141 | 177223931 | GRMZM2G316366 | mads-transcription factor 19, mads19 |
| 5 | 177229408 | 177232968 | GRMZM2G492156 | mads-box transcription factor |
| 5 | 177233625 | 177234595 | GRMZM2G016707 | uncharacterized protein LOC100382421 |
| 5 | 177239242 | 177242092 | AC210598.3_FG003 | phytochelatin synthase |
| 5 | 177442842 | 177451038 | GRMZM2G103152 | riboflavin biosynthesis protein chloroplastic-like |
| 5 | 177485913 | 177487939 | GRMZM2G454702 | dna helicase |
| 5 | 177662568 | 177663536 | GRMZM5G801651 | ctl-like protein ddb_g0274487-like |
| 5 | 177663621 | 177668212 | GRMZM5G896337 | plasma-membrane choline transporter-like protein |
| 5 | 177668908 | 177670329 | GRMZM2G459166 | f-box protein gid2-like |
| 5 | 177739535 | 177743873 | GRMZM2G086246 | dna-(apurinic or apyrimidinic site) lyase |
| 5 | 177745636 | 177749055 | GRMZM5G861269 | microtubule motor |
| 5 | 177824670 | 177827620 | GRMZM2G410710 | prohibitin-mitochondrial-like |
| 5 | 177830186 | 177837302 | GRMZM2G176382 | uncharacterized protein LOC100383781 |
| 5 | 177840203 | 177843844 | GRMZM2G176253 | nitrate transporter |
| 5 | 177973750 | 177974790 | AC212286.3_FG007 | --NA-- |
| 5 | 177975509 | 177977387 | GRMZM2G095861 | potassium channel |
| 5 | 178036903 | 178043555 | GRMZM2G095782 | trna pseudouridine synthase family protein |
| 7 | 37024548 | 37030325 | GRMZM2G025231 | probable cellulose synthase a catalytic subunit 5 |
| 7 | 37254380 | 37258018 | GRMZM2G177539 | folate biopterin transporter family protein |
| 7 | 37726780 | 37727688 | GRMZM5G805008 | BTB/POZ domain protein |
| 7 | 37744688 | 37745329 | GRMZM5G844051 | tetratricopeptide repeat protein 15 |
| 7 | 37834224 | 37847984 | GRMZM2G071877 | protein kinase pti1 |
| 7 | 38025162 | 38027027 | GRMZM2G016622 | photosystem I reaction center subunit IV a |
| 7 | 38182755 | 38183450 | GRMZM2G704490 | --NA-- |
| 7 | 38198939 | 38203333 | GRMZM2G034442 | protein phosphatase 2c 29-like |
| 7 | 38205507 | 38218647 | GRMZM2G034288 | histone-lysine n-h3 lysine-9 specific suvh9 |
| 7 | 38391351 | 38395817 | GRMZM2G140394 | protein binding |
| 7 | 38539990 | 38542053 | GRMZM2G068519 | hypothetical protein |
| 7 | 38601046 | 38603037 | AC213431.4_FG002 | --NA-- |
| 7 | 38619016 | 38620530 | GRMZM2G115957 | pentatricopeptide repeat-containing protein chloroplastic-like |
